# Unmasking Latent Primary Hyperparathyroidism in Guillain-Barré Syndrome: A Case Report and Literature Review

**DOI:** 10.7759/cureus.79161

**Published:** 2025-02-17

**Authors:** Masahiro Biyajima, Hirotaka Oide, Jun Tsuyuzaki, Yuko Kawai

**Affiliations:** 1 Department of Neurology, Asama Nanroku Komoro Medical Center, Komoro, JPN; 2 Department of Diabetes, Endocrinology and Metabolism, Asama Nanroku Komoro Medical Center, Komoro, JPN; 3 Department of Diabetes, Endocrinology and Metabolism, Division of Internal Medicine, Shinshu University School of Medicine, Matsumoto, JPN

**Keywords:** guillain- barré syndrome, immobilization hypercalcemia, latent primary hyperparathyroidism, parathyroid hormone (pth), primary hyperparathyroidism (phpt)

## Abstract

Primary hyperparathyroidism (PHPT), a common endocrine disorder, is often diagnosed in its asymptomatic stage. We report the case of a 79-year-old woman with Guillain-Barré syndrome (GBS) who developed progressive hypercalcemia, initially suspected to be immobilization hypercalcemia (IH). However, the detection of elevated intact parathyroid hormone (PTH) confirmed that the hypercalcemia was not solely due to prolonged immobilization associated with GBS but rather due to previously latent PHPT becoming clinically apparent. Treatment with elcatonin and zoledronate inhibited bone resorption, while cinacalcet suppressed PTH secretion, collectively normalizing serum calcium levels and alleviating symptoms. This case illustrates how prolonged immobilization in GBS can enhance bone resorption, leading to IH and unmasking latent PHPT. Recognizing this mechanism underscores the importance of routine calcium monitoring, PTH screening in high-risk patients, and timely intervention to prevent complications, particularly in immobilized or ageing populations.

## Introduction

Primary hyperparathyroidism (PHPT) is a relatively common endocrine disorder with an estimated prevalence of 0.1% or higher [1]. It results from excessive secretion of parathyroid hormone (PTH), leading to hypercalcemia, which can cause a range of symptoms including fatigue, muscle weakness, neuropsychiatric disturbances, and nephrolithiasis. While many cases of asymptomatic or latent PHPT remain stable, certain conditions, medications, or physiological stressors can trigger overt disease [2]. However, the mechanisms by which latent PHPT transitions to a clinically apparent state remain incompletely understood.

Guillain-Barré syndrome (GBS) is an acute immune-mediated polyneuropathy characterized by progressive muscle weakness and areflexia due to peripheral nerve demyelination or axonal damage. Patients with GBS frequently experience immobilization, a known risk factor for hypercalcemia (immobilization hypercalcemia, IH) due to increased bone resorption. In this report, we describe a rare case in which GBS-associated immobilization led to IH, unmasking latent PHPT and resulting in the clinical manifestation of hypercalcemia. This case highlights the importance of recognizing prolonged immobilization as a potential trigger for the clinical onset of PHPT in patients with previously undiagnosed disease. Early biochemical assessment in immobilized patients may be crucial in identifying underlying endocrine disorders and preventing complications associated with IH and PHPT.

## Case presentation

A 79-year-old woman was admitted to the hospital after a fall resulting in a left fibular fracture. She reported a two-day history of limb numbness and muscle weakness before the fall, which progressively led to difficulty walking. Upon arrival, she was alert, and her vital signs were stable. However, deep tendon reflexes in all limbs were absent, and muscle strength was reduced to a manual muscle test (MMT) grade of 3-4. Grip strength was 7/10 kg. Pathologic reflexes were absent, and glove-and-stocking-type sensory impairment was observed. Based on her clinical course and physical findings, she was diagnosed with GBS, characterized by progressive peripheral neuropathy. She was administered a five-day course of intravenous immunoglobulin (IVIg) therapy and continued rehabilitation.

Blood tests at admission revealed mild hypercalcemia with a corrected calcium level (Payne’s formula) of 11.2 mg/dL (reference range: 8.8-10.1 mg/dL), which worsened progressively during her hospitalization. The laboratory findings at admission are summarized in Table 1.

**Table 1:** Blood test results ^†^ Later confirmed value. WBC: white blood cell; RBC: red blood cell; Hb: hemoglobin; PLT: platelet; PT: prothrombin time; INR: international normalized ratio; APTT: activated partial thromboplastin time; Alb: albumin; Cre: creatinine; BUN: blood urea nitrogen; AST: aspartate aminotransferase; ALT: alanine aminotransferase; LDH: lactate dehydrogenase; Na: sodium; K: potassium; Cl: chloride; Ca: calcium; P: phosphorus; UA: uric acid; CRP: C-reactive protein; PTH: parathyroid hormone; PTHrP: parathyroid hormone-related peptide

Blood count	Value	Reference range	
WBC	10,900	3.300-8,600	/µL
Neutrophil	68.1	38.0-74.0	%
Lymphocyte	25.3	16.5-49.5	%
Monocyte	5.3	2.0-10.0	%
Eosinophil	0.7	1.0-9.0	%
Basophil	0.6	<2.0	%
RBC	465	386-492×10^4^	/µL
Hb	15.1	11.6-14.8	g/dL
PLT	62.9	15.8-34.8×10^4^	/µL
Blood coagulation test			
PT (s)	10.5	9.9-11.8	s
PT (INR)	1.01		
APTT (s)	34.3	23-40	s
D-dimer	1.8	<1.0	μg/mL
Biochemistry			
Alb	4.3	4.1-5.1	g/dL
Cre	0.62	0.46-0.79	mg/dL
BUN	7.7	8-20	mg/dL
AST	26	13-30	U/L
ALT	16	7-23	U/L
LDH	198	124-222	U/L
Na	141	138-145	mEq/L
K	4.2	3.6-4.8	mEq/L
Cl	108	101-108	mEq/L
Ca	11.2	8.8-10.1	mg/dL
P	2.1	2.7-4.6	mg/dL
UA	5.9	2.6-5.5	mg/dL
CRP	0.12	<0.14	mg/dL
Intact PTH^†^	139	10-65	pg/mL
PTHrP^†^	<1.0	<1.1	pmol/L

**Figure 1:**
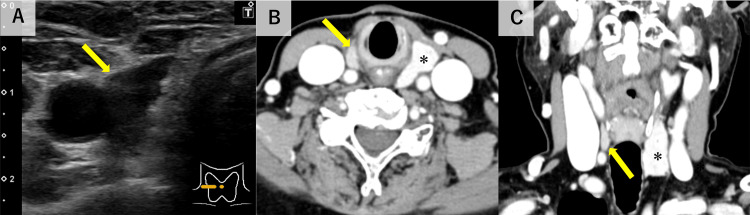
Neck ultrasound and contrast-enhanced computed tomography (A) Neck ultrasound shows an 11×5×11-mm mass to the right side of the trachea (arrow). Contrast-enhanced computed tomography (B: axial view, C: coronal view) shows an enhanced mass on the right side of the trachea (arrows). The asterisk indicates the left lobe of the thyroid gland.

Renal function was normal. She had no history of supplementation with vitamin D or thiazide diuretics, ruling out drug-induced hypercalcemia. Additionally, her fractional excretion of calcium (FeCa) was 6.8% (≥1), excluding familial hypocalciuric hypercalcemia. IH due to immobilization associated with GBS was suspected, and hydration therapy was initiated. Her prolonged immobilization due to GBS was considered a contributing factor to the worsening hypercalcemia. However, her intact PTH level was elevated at 139 pg/mL (reference range: 10-65 pg/mL); parathyroid hormone-related peptide (PTHrP) was negative. The patient had a history of right thyroid lobectomy for a thyroid tumor at the age of 37. Neck ultrasound revealed an 11×5×11-mm mass to the right side of the trachea (Fig. 1A), which was confirmed on contrast-enhanced neck computed tomography (CT) as an enhancing mass in the same location (Fig. 1B, 1C).

**Figure 2:**
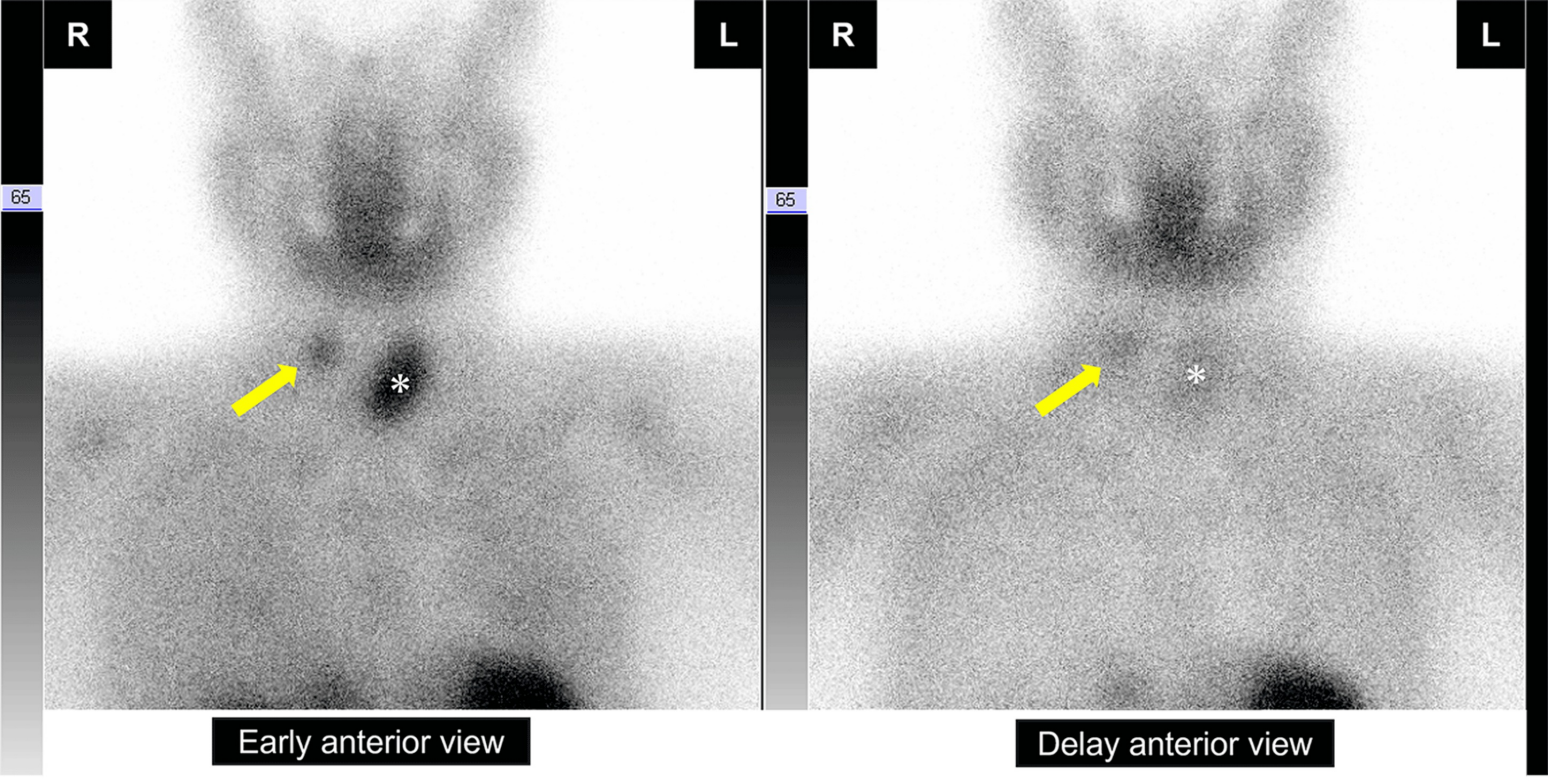
Technetium-99m sestamibi scintigraphy Technetium-99m sestamibi scintigraphy shows increased uptake in the right parathyroid gland (arrow). The asterisk indicates the left lobe of the thyroid gland.

Technetium-99m sestamibi scintigraphy showed tracer accumulation in the right cervical region (Fig. 2), and we diagnosed her with PHPT.

**Figure 3:**
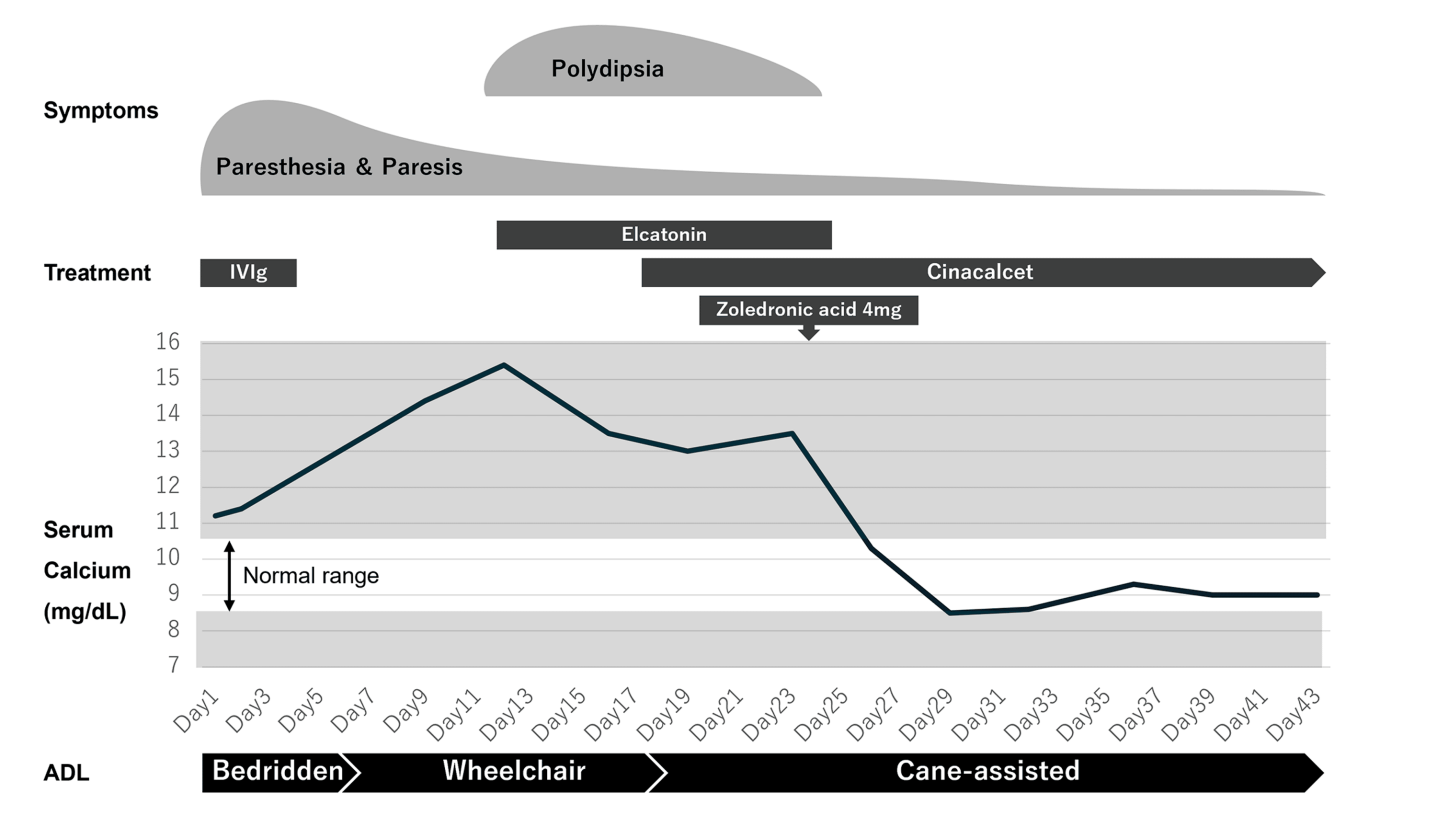
Summary of the clinical course of the patient Paresthesia and paresis improved gradually following IVIg treatment, leading to improved ADL. After hospitalization, serum calcium levels increased gradually, and polydipsia appeared on Day 10. Treatment with elcatonin, cinacalcet, and zoledronate successfully normalized serum calcium levels, resulting in the resolution of polydipsia.

As hypercalcemia worsened due to ongoing PTH overproduction, she developed polydipsia, which resolved following treatment with elcatonin, cinacalcet, and zoledronate (Fig. 3).

**Figure 4:**
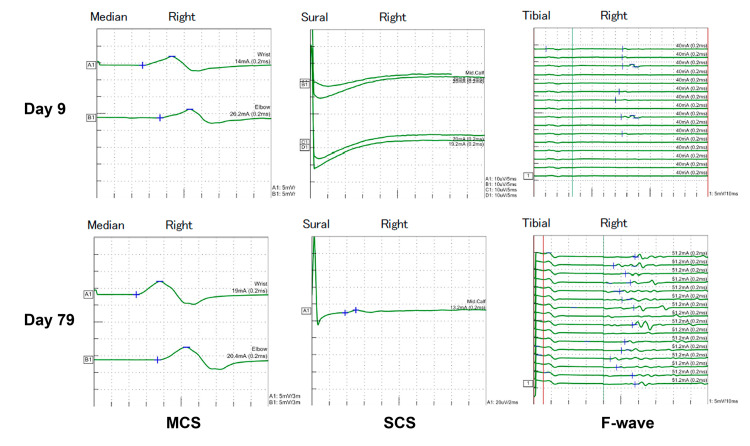
Nerve conduction studies in the acute and recovery phases Nerve conduction studies revealed improved amplitudes of CMAP and SNAP, as well as increased F-wave persistence. CMAP: compound muscle action potentials, MCS: motor conduction study, SCS: sensory conduction study, SNAP: sensory nerve action potentials.

Her muscle strength improved, allowing her to increase mobility progressively, and rehabilitation was continued. Nerve conduction studies revealed improved amplitudes of compound muscle action potentials (CMAP) and sensory nerve action potentials (SNAP), as well as increased F-wave persistence, confirming objective improvement in her peripheral neuropathy (Fig. 4). She was discharged home on Day 62.

## Discussion

Immobilization hypercalcemia

IH was first described by Albright et al. in 1941 [3]. Since then, numerous cases and reviews have been reported, reflecting its clinical importance. While the pathophysiology of IH is incompletely understood, increased bone resorption due to the loss of mechanical stimulation to the skeleton is considered a key factor [4]. The causes of immobilization vary widely, including postoperative states such as abdominal surgery [5] and bariatric surgery [6], and conditions such as stroke [7], traumatic brain injury [8], spinal cord injury [9], burns [10], and COVID-19 [11]. Similar to the present case, two earlier reports have described IH triggered by GBS [12, 13].

In IH, PTH levels are typically reactively suppressed, with hypercalcemia resulting primarily from abnormalities in bone turnover. Hypercalcemia with normal or suppressed PTH levels is classified as parathyroid-independent hypercalcemia, a condition characterized by elevated calcium levels unrelated to primary hyperparathyroidism [14], of which IH is one example. Treatment during immobilization typically involves hydration and administration of elcatonin, bisphosphonates, or denosumab. Once mobility is restored, hypercalcemia often resolves without further intervention. However, in cases of irreversible immobilization, continued supportive therapy may be required.

In the present case, IH due to immobilization associated with GBS was initially suspected; however, elevated intact PTH levels confirmed the diagnosis of PHPT. The prolonged immobilization associated with GBS led to IH, which subsequently unmasked latent PHPT. This case adds to the growing evidence that prolonged immobilization, including that associated with GBS, can serve as a key trigger for unmasking latent PHPT. In such cases, resolution of immobilization alone is insufficient for fundamental improvement, and therapeutic intervention targeting PHPT is necessary.

Latent PHPT

PHPT is a relatively common endocrine disorder and, along with malignancies, is among the frequent causes of hypercalcemia [15]. Malignancy-associated hypercalcemia (MAH) occurs due to mechanisms such as PTHrP secretion, osteolytic bone metastases, or excess calcitriol production, and is a key differential diagnosis of PHPT. In countries with widespread multi-panel biochemical screening, PHPT is often detected early in its asymptomatic stage through routine testing [16]. However, insufficient PTH testing in hypercalcemic patients has been reported, suggesting underdiagnosis of PHPT [17]. Disturbances in calcium homeostasis can manifest several years before clinical diagnosis and latent PHPT is characterized as normocalcemic hyperparathyroidism or normoparathyroid hypercalcemia [18].

In latent PHPT, immobilization can exacerbate hypercalcemia, as observed in the present case. A similar case of PHPT identified following the onset of GBS has been previously reported. Key differentiating features between IH and PHPT include a consistently high PTH level, lack of PTH suppression during the calcium infusion test, and the ineffectiveness of oral etidronate for hypercalcemia management [19].

Latent PHPT cases, along with the triggers leading to their unmasking, are summarized in Table 2.

**Table 2:** Characteristics of the reported cases of latent primary hyperparathyroidism ATRA: all-trans retinoic acid, N/A: not applicable, SGLT-2: sodium-glucose co-transporter 2 The following PubMed search query using search terms was used to identify relevant studies: ("subclinical hyperparathyroidism" OR "asymptomatic hyperparathyroidism" OR "latent hyperparathyroidism" OR "unmask hyperparathyroidism" OR "masquerading hyperparathyroidism") AND (review[pt] OR case reports[pt] OR clinical trial[pt]) NOT (animals[mh] NOT humans[mh]) The search on January 5, 2025, yielded 100 results, of which 15 were selected due to meeting the specified criteria.

Citation	Age/Sex	Trigger for Manifestation	Parathyroid Lesion
Eze et al. (2024) [20]	15/M	Ramadan fasting	Adenoma
Golbus et al. (2023) [21]	73/F	Amiloride (potassium-sparing diuretic)	Adenoma
Akhanlı et al. (2020) [22]	49/M	Dapagliflozin (SGLT-2 inhibitors)	Adenoma
Khan et al. (2017) [16]	17/M	Calcium and vitamin D replacement therapy	Adenoma
Yanamandra et al. (2016) [23]	35/F	ATRA therapy	Adenoma
Bala et al. (2015) [24]	38/F	Vitamin D replacement therapy	Adenoma
Asghar et al. (2012) [25]	55/F	Vitamin D replacement therapy	Adenoma
Hannan et al. (2004) [26]	43/ N/A	Vitamin D replacement therapy	Adenoma
Taylor et al. (1997) [27]	43/F	Vitamin D replacement therapy	Adenoma
Taylor et al. (1997) [27]	54/M	Vitamin D replacement therapy	N/A
Forbes et al. (1995) [28]	88/F	Etidronate and calcium carbonate	N/A
Monno et al. (1993) [19]	38/M	Guillain-Barré Syndrome	Adenoma
Peter et al. (1993) [29]	63/F	L-thyroxine	Adenoma
Hollands et al. (1983) [30]	43/F	Nephrectomy	Adenoma
Graze et al. (1981) [31]	N/A	Lithium therapy	Adenoma

A literature search using PubMed was conducted to identify studies related to subclinical, asymptomatic, latent, unmasked, and masquerading hyperparathyroidism. Among the 100 search results, 15 studies were selected based on relevance and available information [16, 19-31]. Details of the search strategy are provided in the legend of Table 2. This review focuses specifically on cases where asymptomatic or latent PHPT became clinically evident due to specific triggers, excluding cases where PHPT was diagnosed incidentally without prior symptoms. The most common triggers for the unmasking of PHPT were medications, in particular, vitamin D supplementation. Lithium, a well-known cause of drug-induced hyperparathyroidism, can unmask cases of PHPT not caused by the drug itself [31]. Severe PHPT can present as a parathyroid crisis [25], highlighting the importance of PTH measurement and screening for parathyroid tumors, even in cases of mild hypercalcemia. In the reviewed cases, parathyroid adenomas were identified in all patients. While parathyroidectomy is the definitive treatment for PHPT, surgical indications for asymptomatic cases remain controversial. Most patients, including the present case, receive conservative management [32, 33].

With an aging global population, the prevalence of immobilization due to bedridden states is expected to rise. Additionally, the incidence of hip fractures is projected to nearly double between 2018 and 2050 [34], likely resulting in increased immobilization-related conditions. Consequently, cases of PHPT unmasked by immobilization, similar to the present case, are expected to become more frequent. Drug-induced hypercalcemia, serving as a trigger for PHPT diagnosis, will likely persist as a significant clinical concern. Regularly monitoring calcium levels and screening parathyroid function are crucial to prevent severe hypercalcemia. Early therapeutic intervention for patients requiring treatment is essential to ensure optimal outcomes.

Limitations

A limitation of this study is the potential omission of relevant cases due to deficiencies in the PubMed search strategy. The search query may not have captured all instances of unmasked PHPT, given the condition’s prevalence and variability in terminology across studies. Additional triggers beyond those identified in this review may also exist. Further case reports and studies are needed to better define the circumstances under which PHPT becomes unmasked.

## Conclusions

This case illustrates how prolonged immobilization associated with GBS can unmask latent PHPT. Although IH due to prolonged immobilization was initially suspected, persistently elevated PTH levels confirmed the diagnosis of PHPT. The progression of hypercalcemia despite standard IH management necessitated targeted PHPT treatment, emphasizing the need for thorough biochemical evaluation in immobilization-related hypercalcemia. Asymptomatic PHPT is often underdiagnosed, with triggers such as immobilization and medications precipitating its clinical manifestation. Given the rising prevalence of immobilization-related conditions, recognizing latent PHPT and its potential triggers is essential. Routine calcium and PTH monitoring in immobilized patients may enable early detection and timely intervention, preventing complications associated with PHPT and IH.
